# Influence of different fixation modes on biomechanical conduction of 3D printed prostheses for treating critical diaphyseal defects of lower limbs: A finite element study

**DOI:** 10.3389/fsurg.2022.959306

**Published:** 2022-08-24

**Authors:** Bingchuan Liu, Yang Lv, Xingcai Li, Zhongjun Liu, Yufeng Zheng, Peng Wen, Ning Liu, Yaping Huo, Fang Zhou, Yun Tian

**Affiliations:** ^1^ Department of Orthopaedics, Peking University Third Hospital, Beijing, China; ^2^ Engineering Research Center of Bone and Joint Precision Medicine, Ministry of Education, Peking University Third Hospital, Beijing, China; ^3^ School of Materials Science and Engineering, Peking University, Beijing, China; ^4^ Department of Mechanical Engineering, Tsinghua University, Beijing, China; ^5^ R&D Center, AK Medical Co., Ltd., Beijing, China

**Keywords:** 3D printing technology, finite element analysis, diaphyseal bone defect, biomechanical characteristics, fixation mode

## Abstract

**Background:**

Applying 3D printed prostheses to repair diaphyseal defects of lower limbs has been clinically conducted in orthopedics. However, there is still no unified reference standard for which the prosthesis design and fixation mode are more conducive to appropriate biomechanical conduction.

**Methods:**

We built five different types of prosthesis designs and fixation modes, from Mode I to Mode V. Finite element analysis (FEA) was used to study and compare the mechanical environments of overall bone-prosthesis structure, and the maximum stress concentration were recorded. Additionally, by comparing the maximum von Mises stress of bone, intramedullary (IM) nail, screw, and prosthesis with their intrinsic yield strength, the risk of fixation failure was further clarified.

**Results:**

In the modes in which the prosthesis was fixed by an interlocking IM nail (Mode I and Mode IV), the stress mainly concentrated at the distal bone-prosthesis interface and the middle-distal region of nail. When a prosthesis with integrally printed IM nail and lateral wings was implanted (Mode II), the stress mainly concentrated at the bone-prosthesis junctional region. For cases with partially lateral defects, the prosthesis with integrally printed wings mainly played a role in reconstructing the structural integrity of bone, but had a weak role in sharing the stress conduction (Mode V). The maximum von Mises stress of both the proximal and distal tibia appeared in Mode III, which were 18.5 and 47.1 MPa. The maximum peak stress shared by the prosthesis, screws and IM nails appeared in Mode II, III and I, which were 51.8, 87.2, and 101.8 MPa, respectively. These peak stresses were all lower than the yield strength of the materials themselves. Thus, the bending and breakage of both bone and implants were unlikely to happen.

**Conclusion:**

For the application of 3D printed prostheses to repair diaphyseal defects, different fixation modes will lead to the change of biomechanical environment. Interlocking IM nail fixation is beneficial to uniform stress conduction, and conducive to new bone regeneration in the view of biomechanical point. All five modes we established have reliable biomechanical safety.

## Background

The treatment of critical bone defects of lower limbs is a challenge in orthopedics. Bone grafting has always been regarded as one of an effective treatment choice, however, owing to the limited source of bone substitutes, lack of sufficient mechanical strength, and potential risk of transmitting disease and rejection reaction, a preferable defect filling material has been called for (1, 2). Besides, the difficulty of bone defect repair will be further increased when relating to osteomyelitis. Patient-specific implants are alternative for repairing complicated bone defects, which has been applied for limb salvaging since the late 1940’s. With the continuous development of bone tissue engineering, three-dimensional (3D) printed titanium alloy prostheses have been used to repair critical bone defects in various orthopedics fields, including tumor resection (3), osteomyelitis (4), comminuted fracture (5) and revision surgery (6). Compared to traditional therapeutic methods, 3D printed prostheses possess unique advantages in repairing irregular defects, reconstructing biomechanical conduction, and facilitating early rehabilitation exercises (1, 3–7). But the stable fixation of metallic prostheses, the risk of loosening and fracture, and the related stress shielding effect are all key concerns for the clinical repair of bone defects by 3D printed titanium alloy prostheses.

To date, the fixation modes of 3D printed prostheses for treating diaphyseal defects include interlocking intramedullary (IM) nails for hollow cylinder prostheses (8), screws for integrated printed wings (9), and enhanced by an additional lateral osteosynthesis plate (9). However, related studies mainly focus on case reports, lacking of systematic analysis and comprehensive clinical array. Meanwhile, some studies have reported that bone cement was beneficial to enhancing the prosthesis fixation stability, which was more commonly applied in total knee arthroplasty (TKA) and hip arthroplasty (10, 11). But cemented fixation is rarely used to repair diaphyseal bone defects with 3D printed porous prostheses, because the cement block will affect the inner growth of new bone, which is detrimental to the long-term stability of the prosthesis. A recent study has proved that cementless stem with a unique tapered press-fit design showed good short-term in maintaining stable and bond stock, proving cemented fixation is not necessary for prosthesis fixation (12). In addition, the selection of the prosthesis fixation mode mainly depends on doctors' own clinical experience and subjective judgment, but there is currently no unified reference standard for determining the fixation mode. According to Wolff's law, bone can make a continuous adaptive reconstruction to mechanical loading changes and remodel along force conduction (13). Therefore, identifying the appropriate fixation modes that can help biomechanical force to distribute equally around the prostheses not only contributes to early and long-term stability, but also contributes to continuous bone growth.

Finite element analysis (FEA) is a numerical tool for the quantification and simulation of structures and systems, providing an accurate prediction of a component's response subjected to different types of loads and boundary conditions (14). FEA can be utilized for a variety of purposes in orthopedics, which have been previously categorized as either: (1) fundamental understanding, (2) implant design, (3) preoperative planning, or (4) detection of nonunion (15, 16). FEA has been applied to evaluate and compare the biomechanical characteristics in prosthesis design and stem fixation (E,F,G) (17–19).In terms of the design of 3D printed prostheses, FEA has also been applied to analyze biomechanical performance and stress distribution (20–22), and predict the bone-implant interface stress. Wu et al. applied FEA to compare the differences in the mechanical properties of two prosthesis model and successfully designed a 3D printed polyether ether ketone femoral shaft prosthesis for repairing critical bone defects (23). These results have laid a foundation for the development of this study. However, so far, comparative studies have not been conducted on the design and fixation mode of 3D printed titanium alloy prostheses for diaphyseal defects based on FEA.

In this study, we addressed the above problems by conducting a preclinical biomechanical study based on finite element models. For FEA analysis, we designed different forms of prostheses for repairing diaphyseal bone defects, and applied different fixation modes to stabilize the prostheses, so as to analyze the biomechanical distribution characteristics around the prosthesis under different fixation modes. This study aimed to examine the effects of different prosthesis fixation modes on the local biomechanical environment and determine the appropriate fixation mode for different diaphyseal defects from a biomechanical point of view.

## Methods

### Design of a 3D printed custom prosthesis

**Figure 1:**
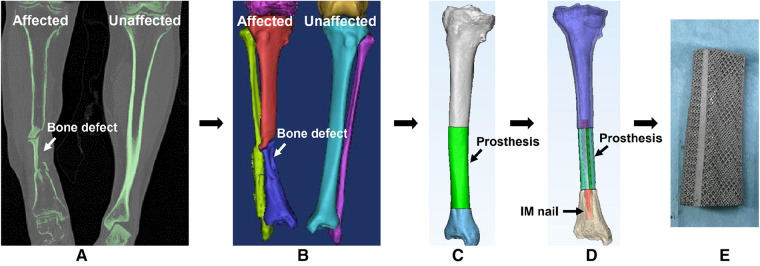
The design process of a 3D printed porous prosthesis. (**A**) CT scan of both the affected and unaffected sides. (**B**) 3D reconstruction was performed based on CT images, then according to the principle of mirror symmetry, the shape of the unaffected bone was converted to the model of the affected side. (**C**) The bone defect was filled by the simulative 3D-printed prosthesis. (**D**) The internal fixation was simulated *via* a medical-industrial interactive platform. (**E**) Finally, the personalized 3D printed porous prosthesis could be fabricated successfully.

As shown in Figure 1, we designed a customized prosthesis using special anatomic landmarks and natural skeletal structures from the unaffected side as shown on bilateral high-precision computed tomography (CT) images. The intact skeleton structure mirrored the disease-affected side, and the defect regions overlapped one another. In light of specific circumstances and objectives, we designed integrally printed structures, including middle cylindrical channels, printed-lateral wings, and printed-IM nails, for the following fixation modes. The “IM nail” refers to a single true rod that passes through the intercalary body and is not integral; and the “printed IM nail” refers to the manufactured nail integral to the prosthesis body as one piece. The “printed lateral wing” refers to the integrally manufactured wing as well.

### Finite element modeling and analysis

**Figure 2:**
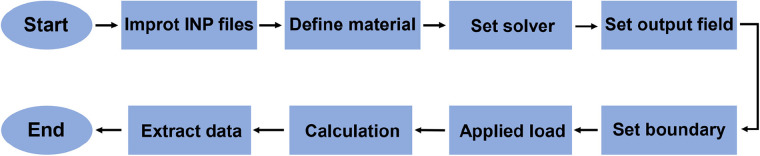
The flowchart of whole simulation analysis process.

We used the critical bone defect of the diaphyseal tibia as an example for the FEA study, and the research results were applicable equally to femur as well. The whole simulation analysis flowchart is presented in Figure 2.

#### Modeling of the tibia, prosthesis, IM nail and screws

The tibia CT image (DICOM format) was imported into the Mimics software (Version 17.0, Materialize, Belgium) to segment bone from the entire model by thresholding. We used the CT Hounsfield unit to construct the cortical and cancellous bone of the tibia, and set the cortical bone thickness from 1.4 to 8.8 mm. The elasticity modulus and Poisson's ratio of cortical and cancellous bone were set as 16,600 MPa, 0.3 and 8,000 MPa, 0.3, respectively. The pore size and porosity of the prosthesis is 640 μm and 70%, and the its wire diameter is 550 μm. The spatial structure and material properties of the prosthesis, IM nail and screws are homogeneous and isotropic. The elasticity modulus and Poisson's ratio of the 3D printed porous Ti6Al4V prosthesis were set as 3,000 MPa and 0.3. The IM nail and screws were identified as the properties of titanium alloy, with an elasticity modulus and Poisson's ratio of 110,000 MPa and 0.3. Surface smoothing, small gap filling and ragged edge removal were performed using UG 1 software (Version 12.0, Siemens PLM Software, USA), after which an STP file was acquired.

#### Meshing

**Figure 3:**
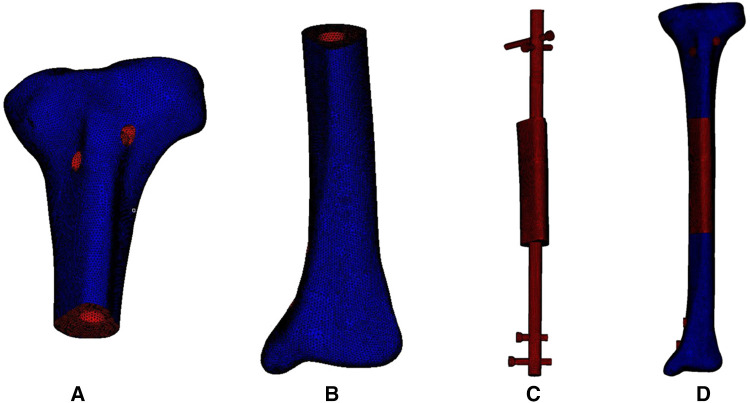
Meshing of the 3D model of the bone, intramedullary nail and prosthesis. (**A**) Meshing model of proximal tibia. (**B**) Meshing model of distal tibia. (**C**) Meshing model of internal fixation and prosthesis. (**D**) Meshing model of the integral structure.

The acquired STP file was imported into HyperMesh 14.0 software (Altair, USA) for geometric cleaning and meshing. The implants were divided into 2 mm tetrahedral meshes, and the bone was divided into 0.2–1 mm tetrahedral and triangular shell element meshes. The triangular meshes simulated the cancellous bone, and the outer shell element meshes simulated the cortical bone. As shown in Figure 3, the triangular meshes are red and the shell element meshes are dark blue. The mesh file was saved in the INP format. Then the INP file was imported into ABAQUS 6.13 software (SIMULIA, Johnston, USA) to build components, which were assigned the corresponding material properties.

#### Boundary, bone-prosthesis contact and stress loading

The prosthesis surface was modelled as an untreated titanium alloy. The contact between bone and prosthesis was modeled as a frictional contact with a friction coefficient of 0.6. A fixed contact was established where the screws were inserted. During the loading stress process, the distal tibia was fully constrained, and a loading force of 700 N along the direction of the force line was applied upon the articular surface of tibial plateau. The stress distribution and maximum von Mises stress were used to evaluate biomechanical concentration characteristics. Besides, the deformation analysis was also utilized to evaluate the stability safety.

## Results

### Different types of prostheses and fixation modes

**Figure 4:**
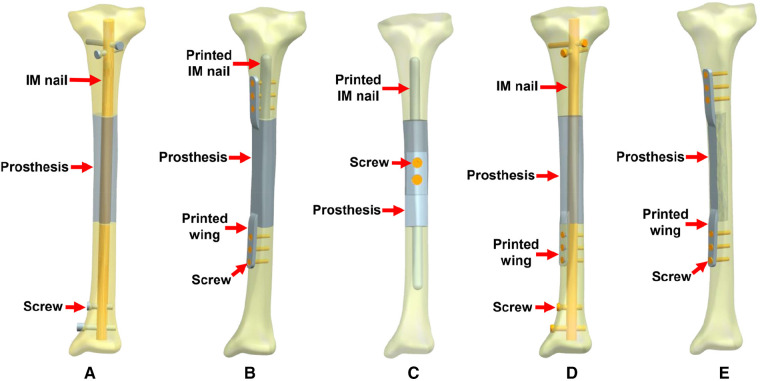
Five types of 3D printed prostheses and fixation modes (IM: intramedullary). (**A**) Mode I: the cylindrical prosthesis was fixed by an interlocking IM nail. (**B**) Mode II: the prosthesis with integrally printed IM nail and wings was fixed by screws. (**C**) Mode III: the assembled prosthesis with integrally printed IM nails was fixed by screws. (**D**) Mode IV: the prosthesis with integrally printed wing was fixed by interlocking IM nail and screws. (**E**) Mode V: the prosthesis with integrally printed wings was fixed by screws.

As shown in Figure 4, according to the location of bone defects and their possible fixation patterns, we established five types of prostheses and fixation modes for the reconstruction of diaphyseal defects. The first four modes were intended to repair the overall complete defect of the diaphysis, while the fifth mode was suitable for the lateral partial defect, in which the lateral bone structure remained intact and continuous.

The characteristics of different prostheses and fixation modes include: (1) Mode I, the simple cylindrical prosthesis is fixed with an interlocking IM nail, due to the elastic fixation property of IM nail, the prosthesis has a certain degree of local micromotion relative to the bone surface; (2) Mode II, the prosthesis has integrally printed IM nail and lateral wings, which can be fixed with bone through screws, with almost no relative micromotion between prosthesis and bone; (3) Mode III, the prosthesis consists of two separate parts, which are fixed by screws in the middle after assembly, the prosthesis has integrally printed IM nails, but the nails are not fixed with screws to promote a wider range of relative micromotion between the prosthesis and bone; (4) Mode IV, the prosthesis has an added an integrally printed lateral wings on the basis of Mode I, with the purpose of increasing the stability of the prosthesis to a certain extent; (5) Mode V, the prosthesis has integrally printed lateral wings that can be fixed with screws for absolute stability.

### FEA modeling and mechanical distribution

The number of nodes and meshes in different FEA models of the five prostheses and fixation modes are listed in Table 1. The different mechanical distributions are displayed as follows, and the maximum stress concentration points are indicated by red arrows.

**Figure 5:**
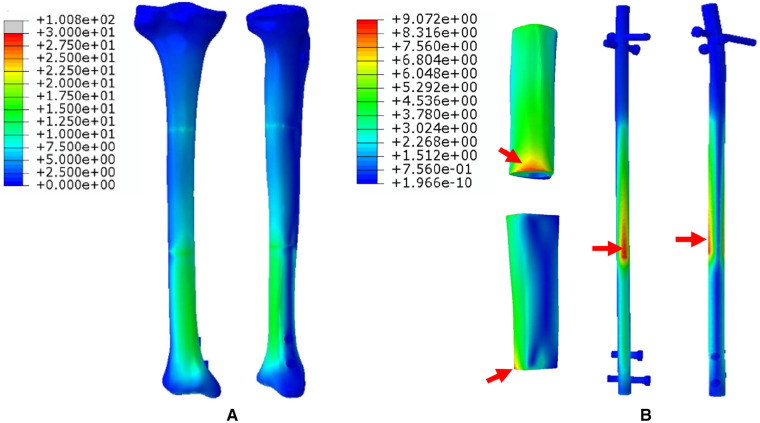
Von Mises stress distribution in Mode I, the red arrows indicate the maximum stress concentration. (**A**) Stress distribution of the integral structure. (**B**) Stress distribution of the prosthesis and internal fixation.

**Figure 6:**
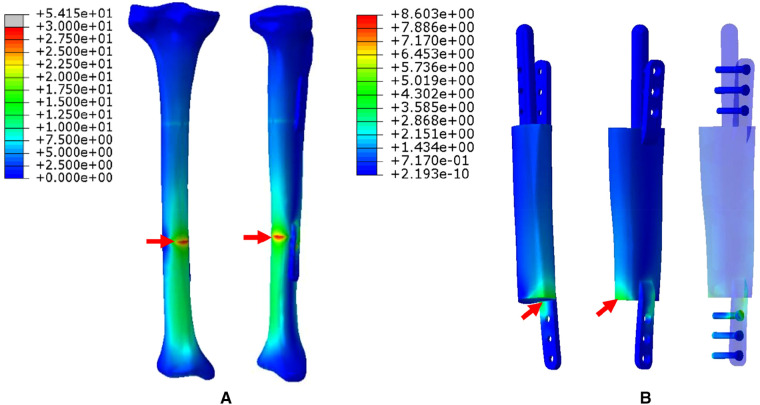
Von Mises stress distribution in Mode II, the red arrows indicate the maximum stress concentration. (**A**) Stress distribution of the integral structure. (**B**) Stress distribution of the prosthesis and internal fixation.

**Figure 7:**
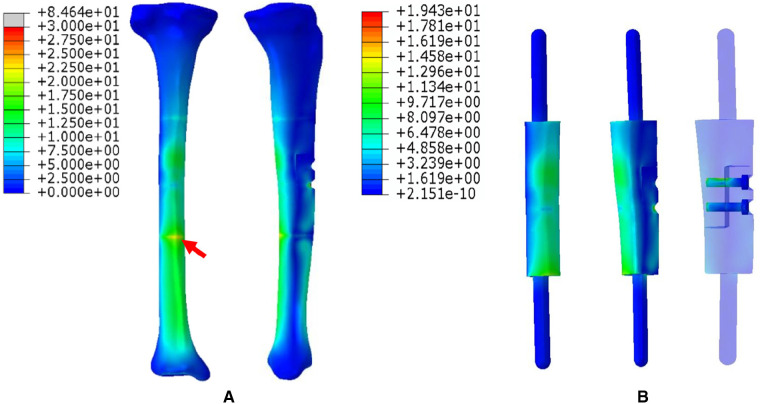
Von Mises stress distribution in Mode III, the red arrows indicate the maximum stress concentration. (**A**) Stress distribution of the integral structure. (**B**) Stress distribution of the prosthesis and internal fixation.

**Figure 8:**
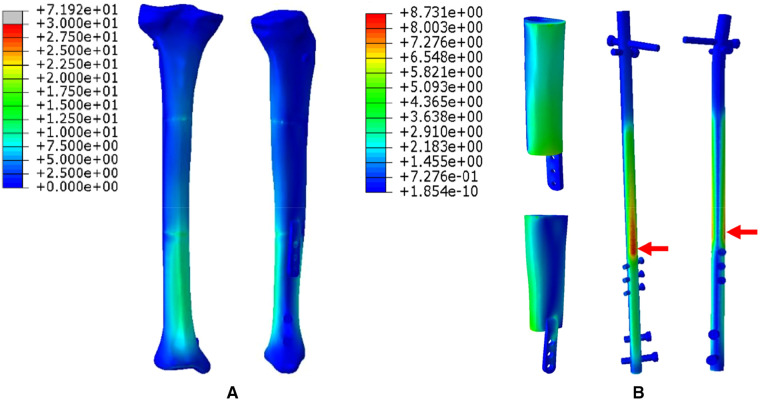
Von Mises stress distribution in Mode IV, the red arrows indicate the maximum stress concentration. (**A**) Stress distribution of the integral structure. (**B**) Stress distribution of the prosthesis and internal fixation.

**Figure 9:**
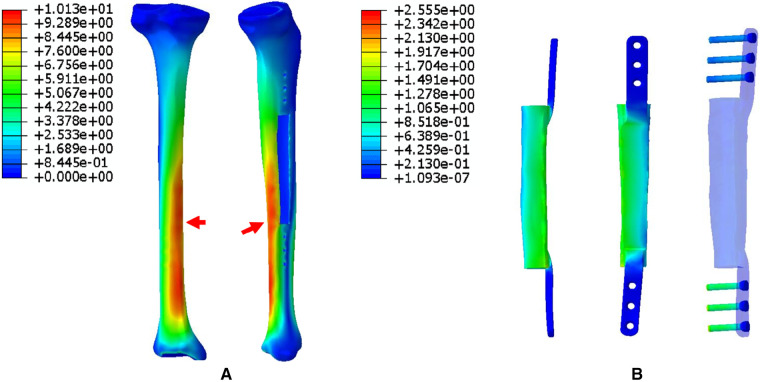
Von Mises stress distribution in Mode V, the red arrows indicate the maximum stress concentration. (**A**) Stress distribution of the integral structure. (**B**) Stress distribution of the prosthesis and internal fixation.

1. The FEA results For Mode I are presented in Figure 5. Figure 5A shows that the stress distribution of the overall bone structure gradually increased from the proximal to distal part. For the implants, we observed that the highest stress distribution was concentrated at the distal end of the prosthesis and middle part of the IM nail (Figure 5B). The stress distribution at the distal end of IM nail was also stronger than that at the proximal end. The stress intensity shared by all four screws was weaker than that shared by the main IM nail.
2. The FEA results for Mode II are presented in Figure 6. The stress distribution intensity at the distal part was higher than that at the proximal part, and the maximum stress concentration was located at the interface between the distal prosthesis and defect end (Figure 6A). The stress distribution on the prosthesis was also mainly concentrated at the distal end, and the stress intensity shared by the three distal screws was greater than that of the three proximal screws (Figure 6B).
3. The FEA results For Mode III are presented in Figure 7. Compared with Mode I and Mode II, the stress distribution in the entire bone structure was more uniform, and the stress-sharing effect and function of the prosthesis were also more obvious, with the maximum stress concentrating at the distal bone-prosthesis interface. The stress shared by the integrally printed IM nails was also weaker, and the stress shared by the proximal screw was higher than that of the distal screw (Figure 7B).
4. The FEA results for Mode IV are presented in Figure 8. Compared to Mode I, this fixation mode with the addition of a distal lateral wing did not significantly change the stress distribution on the overall bone structure. However, the stress distribution on the prosthesis became relatively more uniform, with no evident stress concentration arears. The stress distribution of the IM nail was mainly concentrated at the mid-segment region.
5. The FEA results for Mode V are presented in Figure 9. This is a special case of a diaphyseal defect in which a continuous bone structure remained on one side of the diaphysis. We observed that stress was mainly conducted along the continuous bone structure and was concentrated in the mid-distal region. The stress shared by the prosthesis was weaker than that in the bone structure. The main body of the prosthesis shared more stress than the integrally printed wing, and the three distal screws shared more stress than the proximal three.

**Table 1:** Number of nodes and meshes in different modes.

Fixation mode	Number		
	Nodes	Tetrahedral meshes	Triangular shell meshes
Mode I	170,664	806,174	63,381
Mode II	148,010	774,371	63,080
Mode III	153,430	737,086	63,480
Mode IV	177,110	830,038	63,326
Mode V	165,508	781,668	82,861

### Deformation analysis

**Figure 10:**
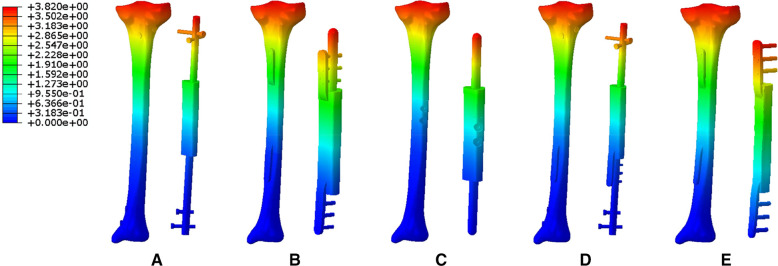
The deformation displacement characteristics of five modes (**A**: Mode I; **B**: Mode II; **C**: Mode III; **D**: Mode IV; **E**: Mode V).

Figure 10 showed different deformation displacement characteristics of five modes under the same external stress environment. From Mode I to Mode V, the maximum displacement values of tibial structure were 3.82, 5.13, 6.30, 2.88, and 1.27 mm; the maximum displacement values of implants were 3.58, 3.99, 4.77, 2.70, and 0.91 mm.

### The maximum von Mises value of bone and implants

**Figure 11:**
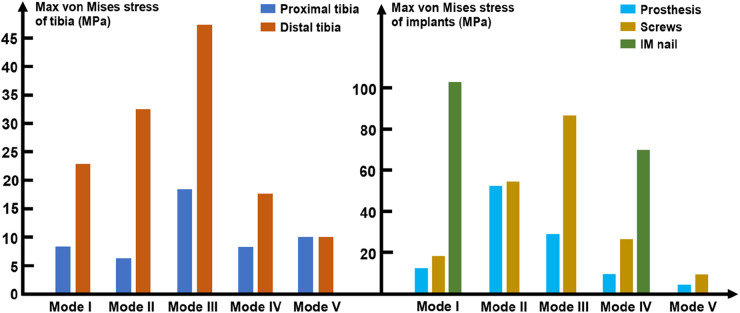
Diagram of the maximum von Mises stress of the tibia and implants (IM: intramedullary).

As mentioned above, loading stress was applied to the tibial plateau and transmitted from the proximal tibia to the distal tibia. Figure 11 shows that the maximum von Mises values of the distal tibia were higher than that those of the proximal tibia. Both the proximal and distal tibia von Mises maxima appeared in Mode III, which were 18.5 and 47.1 MPa, respectively. The IM nail was introduced in Modes I and IV, where the maximum stress on the distal tibia was reduced compared to that in Modes II and III. In Mode V, the proximal and distal tibia were subjected to the same maximum stress of 9.8 MPa.

In terms of the implants, as shown in Figure 11, the maximum peak stress shared by the prosthesis and screws appeared in Mode II and III, which were 51.8 and 87.2 MPa, respectively. The minimum peak stress shared by the prosthesis and screws both appeared in Mode V, which were 4.8 and 10.2 MPa, respectively. In the models with the introduction of IM nail, the maximum peak stress shared by IM nails were much higher than that of the prosthesis and screws, which were 101.8 and 69.7 MPa in Mode I and IV, respectively.

## Discussion

The application of 3D printed porous titanium alloy prostheses to reconstruct critical diaphyseal defects of the lower limbs is a reliable therapeutic option (1). In this study, using the FEA method, we simulated the stress conduction and distribution characteristics of prostheses and fixation modes for treating diaphyseal defects, and confirmed the maximum von Mises concentration regions. These results provide an important reference for clinical decision-making and the choice of prosthesis and fixation mode.

The main requirements to be met for the application of 3D printed titanium alloy prosthesis to repair diaphyseal defects include: (1) the contour and shape of a prosthesis should match the bone defect, which can be achieved by the mirror symmetry effect as shown in Figure 1; (2) the mechanical strength of a prosthesis can meet the requirements of bone biological stress transmission, which can be adjusted by changing the pore size and porosity of the prosthesis (24, 25); (3) appropriate fixation mode, which should guarantee initial and long-term stability, does not affect new bone regeneration, and does not produce stress shielding; and (4) the stress shared by the internal fixation implants should not cause itself to loosen and break, ensuring the safety of lower extremity weight-bearing and functional exercise.

To explore the biomechanical distribution law under different fixation modes, we constructed FEA on five prosthesis models in this study, which can be applied to partial and complete defects of the diaphysis. The establishment of these five models were based on real clinical cases. The prostheses designed in this study were printed as a whole at one time, press-fit IM stem and bone cement were not applied during the insertion and fixation of the prostheses. According to the results, when the prosthesis was fixed with an interlocking IM nail alone, the overall stress distribution was mainly concentrated in the distal region, and the maximum von Mises concentration area of the prosthesis and the IM nail both appeared at the distal junction region between the prosthesis and bone (Mode I, Figure 5). When an integrally printed lateral wing was added, part of the stress was shared by the wing, resulting in stress concentration on the main body of the prosthesis being attenuated (Mode IV, Figure 8). However, the addition of the lateral wing did not significantly affect the stress concentration characteristics of the IM nail when by comparing Mode I and Mode IV. In a previous study, Wu et al. applied a 3D printed polyether ether ketone prosthesis to repair the femoral shaft defect and observed that the stress was concentrated in the middle (23). Similarly, when an IM is used to fix long bone fractures, the stress can also be effectively transmitted and concentrated mainly in the middle region of the IM nail, which could help increase the mechanical stability and reduce stress shielding (26, 27). Our findings are consistent with those of previous studies, suggesting the successful establishment of an effective and reliable model in this study. For the prosthesis with integrally printed IM nails and lateral wings, the stability between the prosthesis and bone surface was stronger than that of the prosthesis fixed with the IM nail after local absolute stability was achieved with screw fixation. At this point, the stress was mainly concentrated in the cross-sectional area between the distal prosthesis and bone, and the stress shared by the prosthesis and screws also concentrated on the distal region (Mode II, Figure 6). If the integrally printed IM nail was not fixed with screws, as in Mode III, then the prosthesis had maximum motion relative to the bone, resulting in the integrally printed IM nail sharing weak stress (Mode III, Figure 7). For Mode V, after the prosthesis was implanted and fixed with screws, the stress still mainly conducted along the lateral continuous bone structure and concentrated in the mid-distal region, and the stress transfer effect of the prosthesis was weak (Figure 9).

The maximum stress is an important indicator for evaluating the bearing safety of implants. If the maximum stress exceeds the yield strength of the implants, they will bend or even break. According to the literature, the longitudinal tension and compression strength of compact bone were nearly 135 ± 15.6 and 205 ± 17.3 MPa, and the transverse tension, compression and shear strength of compact bone were nearly 53 ± 10.7, 131 ± 20.7, and 65 ± 4.0 MPa (28). As shown in Figure 11, in our study, the maximum stress on the bone was in the distal tibia of Mode III. The yield strength parameters of the bone were all greater than the maximum stress borne by the bone after prosthesis implantation, so the bone would not bend or break due to limb weight-bearing. In the modes with additional IM nails, namely Mode I and Mode IV, the stress on the proximal and distal tibia was significantly reduced, but the stress on the IM nails was relatively high, which could reach 101.8 MPa. The yield strength of the titanium alloy ranged from 951 to 1068 MPa (29), which was significantly higher than 101.8 MPa, so there was no risk of bending or breaking of the IM nail at this time. In the modes without additional IM nails, the higher von Mises stress shared by screws was in Mode III, which was 87.2 MPa, significantly lower than the yield strength of the titanium alloy. The maximum von Mises stress of the prosthesis appeared in Mode II, which was significantly lower than the yield strength of titanium alloy as well. In Mode V, the stress distribution of the overall structure was closer to the natural characteristics, with a relatively uniform distribution of proximal and distal regions without an obvious stress concentration area, and the stress on the prosthesis and screws was relatively weak.

Different prosthesis modes can influence subsequent bone reconstruction processes. The gradual regeneration of new bone and the formation of stable osseointegration are the ideal states after the prosthesis implantation for treating bone defects. Local biomechanical environment is an important factor that affect the regeneration and remodeling of new bone (13, 30). Additionally, studies have demonstrated that less rigid fixation and comparable motion improved bone healing, and interfragmentary micromotion was thought to be a prerequisite for healthy fracture union (31, 32). The same notion applies to bone growth after prosthesis implantation inside a bone defect. Interlocking IM nail fixation has the advantage of conforming to biomechanical conduction of the long bone, with a long force arm, and the force line is also close to normal bone. In addition, the elastic fixation of IM nails is also conducive to the formation of a certain degree of mutual micromotion between the prosthesis and bone surface. Under these circumstances, Mode I can theoretically promote new bone growth at both the proximal and distal prosthesis-bone interfaces. For Mode IV with the addition of a lateral wing at the distal prosthesis end, the micromotion effect at the proximal end became stronger; therefore, the rate of new bone growth at the proximal end might be stronger than at the distal end. In Mode II, the prosthesis is fixed firmly by screws, resulting in few local micromotions and creating a negative effect on new bone regeneration. For Mode III in which the prosthesis and bone are not fixed, the integrally printed IM nails swing significantly in the medullary cavity, resulting in relatively larger motion at the interface between the prosthesis and bone, which is not conducive to new bone regeneration and stabilization. In Mode V, less stress is conducted on the prosthesis, resulting in a lack of stimulation for new bone growth on the surface and inside the prosthesis, which is also not conducive to the growth of new bone. Some studies have shown that modelling based on different bones can evaluate the effect of prosthesis stabilization under different bone strengths, and time-dependent FEA model can simulate the effect on new bone reconstruction (33, 34), which is very important to evaluate the dynamic and long-term stability of the prosthesis. But for our present study, since the thickness and regularity of bone growth after prosthesis implantation are not clear, so it is difficult to establish time-dependent model to show how bone remodelling and its impact on long-term stability.

According to the literature, the current application of titanium alloy prostheses to repair bone defects is still mainly in the field of repairing tumor-related defects. The corresponding experiences of success and failure is relatively rich and worth reference. The failure or reoperation rates of tumor endoprostheses range from 24.5% to 49% (35–38), and reoperation for mechanical endoprosthetic events occurred in 33%–49% patients (35, 36). Thus, the biomechanical environment clearly plays an important role in the success of prosthesis implantation. Based on the FEA results of this study, we propose innovative prosthesis design and internal fixation strategies based on the principle of biomechanics for the repair of bone defects *via* 3D printed titanium alloy prosthesis implantation. For simple diaphyseal critical bone defects, it is recommended to choose a cylindrical prosthesis accompanied by interlocking IM nail fixation, such as Mode I and Mode IV, which can not only meet the needs of prosthesis stability and biological force transmission, but also retain the moderate micromotion between the prosthesis and bone surface to promote new bone regeneration. In some cases, such as severe osteoporosis resulting in insufficient holding force of screws, adjacent joint infection or after joint replacement, and patients with narrow medullary cavity or large long bone curvature, IM nail fixation is not applicable, since IM nail placement is difficult. Under these circumstances, prosthesis design and a fixation mode like Mode II is recommended. The stable fixation of the prosthesis can be achieved by screw fixation, and the integrity of bone structure and biomechanical conduction stability can also be reconstructed. For cases with partial lateral defects, Mode V can be applied. Mode III is not recommended for clinical application, because this fixation mode results in high instability of the prosthesis and is not conducive to the transmission of mechanical force, resulting in a high risk of prosthesis loosening and displacement. It is noting that the extra-cortical plate may influence local periosteal blood supply, hence this factor should be considered before choosing later wing fixation.

The main limitation of this study is that valid biological research evidence to corroborate FEA results is still lacking. Further in-depth animal research and clinical follow-up will be helpful to clarify the definite effects of different biomechanical environments on prosthesis stability and new bone growth.

## Conclusion

For the application of 3D printed titanium alloy prostheses to repair critical diaphyseal bone defects of lower limbs, our FEA results show that different prosthesis designs and fixation modes could cause different biomechanical distributions. The additional interlocking IM nail fixation was beneficial to the conduction of biological force and the micromotion of the bone-prosthesis interface, which has the potential to promote new bone regeneration and is recommended for clinical application. For prostheses with an integrally printed IM nail and lateral wings, screw fixation was selected, which was also suitable for some cases that are not suitable for fixation by interlocking IM nails, especially the fixation close to the articular side of metaphysis. For cases with partially lateral bone defects, a prosthesis with integrally printed wings mainly played a role in reconstructing the structural integrity of the bone, but had a weak role in sharing the stress conduction. These study results can provide a certain degree of reference for clinical decision-making and further related in-depth research.

## Data Availability

The raw data supporting the conclusions of this article will be made available by the authors, without undue reservation.
